# Is Frequency of Practice of Different Types of Physical Activity Associated with Health and a Healthy Lifestyle at Different Ages?

**DOI:** 10.3390/ejihpe14010017

**Published:** 2024-01-22

**Authors:** Liudmila Liutsko, Sergey Leonov, Alexander Pashenko, Irina Polikanova

**Affiliations:** 1 ISAN, International Society of Applied Neuropsychology, 08787 La Pobla de Claramunt, Spain; 2 IDIAP JGol, 08007 Barcelona, Spain; 3 Federal Scientific Centre for Psychological and Interdisciplinary Research, Moscow 125009, Russia; svleonov@gmail.com (S.L.); pashchenkoak@mgppu.ru (A.P.)

**Keywords:** healthy lifestyle, physical activity, doctor’s visits, moderate- to high-intensity sports, low- to moderate-intensity sports, leisure activity

## Abstract

Physical activity (PA) has been shown to be related to physical and mental health. Yet there are few studies on how the frequency of PA relates to health and a healthy lifestyle. We aimed to investigate how the frequency of different PAs is associated with the following health indicators: body mass index (BMI), substance consumption, physical health, and mental health. We focused on three types of PA: (1) medium- to high-intensity aerobic exercise; (2) low- to medium-intensity relaxing exercise; and (3) outdoor leisure PA. A total of 9617 volunteers, aged 19 to 81, participated in the study. The relationships between the frequencies of the three types of PA and health-related and sociodemographic factors were analyzed using multinomial logistic regression. We found that women more frequently engaged in PA type 2, and men in types 1 and 3. A higher frequency of PA was associated with lower BMI and less or no smoking behavior; higher education (PAs 1 and 3); higher age (PAs 2 and 3); better physical health (PAs 1 and 3); and better mental health (PA 3). In conclusion, higher frequency of different PAs was significantly associated with better physical and mental health, less smoking, higher age, and a higher level of education.

## 1. Introduction

Physical activity (PA) is a wide range of activities that increase energy expenditure by moving the body [1,2]. Some researchers point to the importance of covering different domains of PA instead of referring only to sport and exercise. These domains are walking, cycling, leisure time, housework, transportation, and occupational PA [3,4,5,6,7,8]. One in four adults globally do not meet the recommended levels of PA [9]. Regular PA has been proven to help prevent and manage non-communicable diseases such as heart disease, stroke, diabetes, several cancers, and hypertension; it also aids in the maintenance of a healthy body weight (normal BMI (body mass index)) [10,11,12]; and can improve mental health [13,14,15], quality of life, and well-being [9,16,17,18,19,20,21]. PA has been reported as an effective adjuvant treatment in major depressive disorder [22]. Aerobic exercises improve functioning parameters and mood in depressed patients [16,17]. Yoga-type, stretching, and resistance training exercises reduce anxiety levels [23,24,25] and maintain cognitive function [6,26,27]; and adequate levels of PA result in emotional well-being and increased energy [7].

Older adults commonly engage in outdoor leisure activities and low-intensity physical activity such as gardening [28,29,30,31,32]. These activities have positive effects on physical and mental health [29,33,34,35,36,37], including self-esteem, self-motivation, willpower, and problem-solving abilities [38,39]. Higher education levels are associated with increased leisure time physical activity [40]. Active older adults have better health outcomes and visit doctors less frequently [36,37,38,41].

PA is classified into different levels, most often high (e.g., jogging, swimming, training at a gym, and tennis), moderate (e.g., daily walking for at least 1 h), and low [4,5]. Norton and colleagues [42] offered a distinct classification of PA into sedentary, light, moderate, vigorous, and high [42]. The Youth Compendium [3] provided a list of 196 physical activities categorized by activity types, specific activities, and metabolic costs.

For the purpose of our study, we focused on several levels and types of PA: (1) medium- to high-intensity exercises (running, aerobics, swimming) (PA type 1), (2) low- to medium-intensity relaxation and stretching exercises (e.g., yoga, tai chi, stretching, Pilates, etc.) (PA type 2), and (3) outdoor leisure group sport and other PAs (gardening) (PA type 3). These physical activities were categorized according to [43], the aim of which was to explore the emotional state of participants and determine whether different types of PA could moderate emotional state. For example, PA type 1 reduced depressive states [23]; PA type 2 reduced anxiety [24]; and PA type 3 could do both and also increase life satisfaction [44].

Smoking alone is related to a range of health problems, including mood and anxiety disorders [1], earlier aging, and death [6]. Along with PA, it is seen as one of the most important lifestyle factors in terms of preventing chronic diseases [6,9]. The relationship between alcohol and PA is controversial, with some studies showing a positive association due to social events like celebrations after competitions [45,46,47,48].

In 2021, within the Eurobarometer project, data from a cross-sectional survey conducted across 28 European countries to determine the prevalence of physical inactivity among adults aged 18–64 were analyzed [49]. The results showed that more than one in three adults in the EU were physically inactive, with significant differences noted across countries. Women, older adults, and those with lower socioeconomic status were less likely to engage in physical activity. Belgium occupied a middle position in physical activity performance [49]. Hence, it is more fitting to scrutinize epidemiological data that pertain to a specific country, rather than relying on a broad European sample [36]. This study focuses on the epidemiological data on the Belgian population.

### 1.1. General Aim of This Study

The aim of this study is to investigate if the frequency of practicing three different types of PA (dependent variables) is associated (both positively and negatively) with healthy lifestyle, consumption habits (smoking and alcohol), BMI, level of study, age, sex, the number of doctor’s visits (except to psychiatrists), and visits to psychiatrists (the latter two being used as indicators of physical and mental health), with these data obtained retrospectively from the health registries for the 10 years preceding the rest of the data collection (independent variables).

### 1.2. Main Hypotheses

A higher frequency of PA type 1 (H1), type 2 (H2), and type 3 (H3) is positively associated with (1) health indicators (obtained retrospectively from the health registries for the 10 years preceding the rest of the data collection), meaning better physical and mental health (namely, a lower number of visits to doctors); and (2) a healthy lifestyle, namely lower BMI, possibly higher alcohol consumption (though this is not related to a healthy lifestyle, but was reported in previous studies, and we aim to investigate it), and less tobacco consumption.

Additionally, we would like to explore the associations of the frequency of engaging in the three types of PA with age and sex due to contradictory data in the literature.

## 2. Materials and Methods

### 2.1. Study Design, Participants, and Original Data Collection

This study has a mixed design; it is cross-sectional and retrospective (we incorporated longitudinal data from health registries). Specifically, we collected records of doctor’s visits and visits to psychiatrists from a 10-year period leading up to the study [43]. This study was based on the general Belgian population with 10,661 participants (see Appendix A for the flowchart of the samples from the original and current studies). The final dataset for analysis consisted of 9617 participants (aged from 19 to 81 years old; M = 56.5, SD = 14, 59% female) after merging those who replied to the survey (PA and healthy lifestyle) with the available health records.

This study received approval from the Ethical Committee of the Psychological Science Department and from the collaborative entity Mutualité Chrétienne-Christelijke Mutualiteit (MC-CM), the law service of the Mutual Benefit Society, and received their financial and organizational help. All respondents received detailed information about the study and the data processing and provided separate written consent for the questionnaire study and the coupling of their answers with their medical records that were in possession of the MC-CM [43]. Only people who provided written consent for both parts were included in the study. The realization of this study (planning of the hypothesis and data analysis) was supported by the MC-CM and by the 2012 Belgian–American Education Foundation (BAEF) alumni award granted to M.M. The MC-CM helped with data collection by providing us with the email addresses of their members and the health records of people with whose consent we coupled survey answers with health records. The preliminary results of this study were presented at the ISEE (International Society for Environmental Epidemiology) conference [50].

### 2.2. Variables

The demographic data (age, sex, and level of study) and epidemiological data such as indicators of physical and mental (the sum of numbers (N) of visits to GPs and all doctors except psychiatrists) (the sum of numbers (N) of visits to psychiatrists) health were taken over the 10 years preceding the original study [43]. Other health and healthy lifestyle factors such as BMI and smoking and alcohol consumption habits were collected together with the questionnaire about the three types of physical activities: 1 (sport): medium- to high-intensity exercises such as aerobics, running, biking, swimming, Nordic walking, etc.; 2 (yoga-type stretching exercises): yoga, tai chi, Pilates, etc.; and 3: outdoor leisure group sports and other PA (gardening) (see Appendix B and Appendix C for more details).

### 2.3. Data Analysis

The descriptive statistics and multinomial logistic regression analysis were performed using STATA v.14. The outcome variables were the frequencies of practicing the three types of PA and the independent variables were the indicators of health and a healthy lifestyle, such as body mass index (BMI), smoking and alcohol consumption, physical health (number of doctor visits excluding to psychiatrists), and mental health (number of visits to psychiatrists), and sociodemographic variables (age, sex, and level of education). The frequencies of practicing the three types of PA, substance consumption, and level of education were categorized before data collection (Appendix B). For the variables that were not categorized during data collection as per the designed survey, such as age, BMI, and the sum of visits to psychiatrists, we used the categories represented in Appendix C and Table 1. For the variable for the physical health indicator, the sum of all doctor’s visits (except to psychiatrists), we used a 25% quartile distribution (Table 1).

## 3. Results

The descriptive statistics of the study variables are presented in Table 1.

The descriptive statistics for sex subgroups are represented in Appendix C (Table A1 for women, Table A2 for men). All study variables differed for both sex subgroups at a statistically significant level, *p* < 0.001 (except PA type 1—intensive to medium sport exercises—where the *p*-value was 0.015, but also significant), measured as per bivariate analysis (chi-square).

Multinomial logistic regression analysis was performed in order to observe where (and in what magnitude) a significant relationship between the three types of physical activities and observed variables of a healthy lifestyle (BMI, health indicators, and substance consumption habits—smoking and alcohol) exists. For general (physical) health indicators, the sum of the number of all doctor’s visits, except to psychiatrists and for mental health, and the sum of the number of visits to psychiatrists from the clinical histories were used as proxies. The results, expressed in terms of relative risk ratios (RRRs) and 95% confidence intervals (CIs), are shown for the whole group, and for women and men for each type of PA: PA type 1 (Table 2); PA type 2 (Table 3); and PA type 3 (Table 4).

Additional regression analysis also showed that the physical health indicators of the participants of this study have a direct relationship with their age and an inverse relationship with their level of education. In contrast, mental health indicators have an inverse relationship with the age of the participants and a direct relationship with their level of education.

Men were more prone to practicing PA type 1 sport activities at the “almost always” level compared with women, with a direct relationship with the variable of sex (men were coded with a higher number; see Appendix B), unlike women (Table 2). BMI was reversely associated with practicing PA type 1 (more frequent practice = lower BMI) (Table 2).

Level of study (in women specifically) played a significant role in the relationship with PA type 1, with a direct relationship indicated between level of study and the frequency of practicing PA type 1 (Table 2).

A higher frequency of all doctors’ visits except to psychiatrists (indicator of physical health) in women was significantly associated with less frequent practice of PA type 1. A weaker and inverse relationship was shown with the frequency of psychiatrist visits (the indicator of mental health) with this PA. In the reverse direction, higher frequency of PA type 1 was associated with better physical health indicators (i.e., fewer visits to doctors except psychiatrists) and with a tendency to have higher mental health scores (i.e., fewer visits to psychiatrists), but only at the level of group values if comparing “almost always” with the baseline, “almost never” (*p* = 0.046) (Table 2).

As for consumption habits, more frequent smoking was associated with less frequent PA type 1, whereas no significant relationship was observed with alcohol consumption (Table 2).

Similar to the PA type 1 results, trends in BMI (reverse), especially in women, and in age and educational level (direct) were observed in the relationship with PA type 2—sport (stretching exercises, low to medium intensity, yoga type) (Table 3). The sex variable showed the inverse of the frequency of PA type 1 association, indicating more frequent practice in women than men (Table 3).

A weak relationship with the indicator of mental health (the sum of the number of visits to psychiatrists) was observed at the whole group level when comparing practice “sometimes” of PA type 2 with the “almost never” baseline (Table 3). More frequent smoking was associated with less frequent practicing of stretching and relaxation exercises in both men and women (Table 3).

Finally, for PA type 3, outdoor leisure group sport and other physical activities (gardening), similar trends were shown to those for PA type 1 in the relationships with sex (men were prone to practicing it more frequently), age, and educational level (direct relationships with higher age and more frequent practice and reverse relationships with BMI and smoking (Table 4)).

Both proxies of general and mental health indicators are associated with higher frequencies of practicing PA type 3 since a reverse relationship is observed between N of doctors’ visits and frequency of practice for the whole group, as well as separately for men and women (Table 4).

Regarding the role association of factors in our study, we found that a higher frequency of practicing type 1 PA was primarily associated with lower BMI (a protective factor; 47% probability of occurrence if practicing “almost always” and 24% for “sometimes” compared with the baseline, “almost never”) and less or no smoking behavior (a protective factor; 41% probability of occurrence with practicing “almost always” and 29% for “sometimes” compared with the baseline, “almost never”). Other important factors included having a higher level of education (a protective factor; 14% and 7% probability of occurrence with practicing “almost always” and “sometimes”, respectively); better physical health (measured by the number of all doctor’s visits, excluding to psychiatrists) (a risk factor; 13% and 9% of occurrence for the categories with practicing PA 1 “almost always” and “sometimes”); mental health, expressed inversely by the sum of the number of visits to psychiatrists (a risk factor; 9% occurrence of having more visits and only significant with practicing PA type 1 “almost always” vs. the baseline “almost never”); and higher alcohol consumption (7% occurrence, but it had a marginal association (*p* < 0.10) for the whole group with practicing “almost always“, and was significant only in the women’s subgroup for the “almost always” category) (Table 2).

A higher frequency of type 3 PA was also found to be associated with similar factors, the most significant being a greater likelihood of a lower BMI; the second most significant factor was older age, the third most significant factor was nonsmoking behavior, and the fourth most significant factor was physical and mental health (Table 4).

Frequency of PA type 2 was not found to be associated with physical health, but positively correlated with poor mental health (Table 3). Moreover, while men showed a greater preference for practicing PA types 3 and 1 than women, women showed a greater trend of practicing PA type 2 more frequently (Table 2, Table 3 and Table 4).

The summary of the results regarding the relevant factors associated with higher frequencies in practicing (“almost always” vs. the baseline, “almost never”) the three PA types is represented in Table 5.

## 4. Discussion

### 4.1. General Discussion

These findings support existing research on the positive impact of PA on reducing the number of doctor visits [51]. These data are needed to better tailor further interventions in order to better formulate public health strategies. The novelty of this study is its investigation of the relationships of the frequency of different types of physical activities with public health (physical and mental). The majority of previous studies reported only on statistically significant associations of changes in increasing PA related only to increasing physical health [52].

This study both confirms and expands upon the existing literature, while also highlighting some discrepancies that require further investigation. Specifically, our findings support the notion that moderate- to high-intensity PA is associated with lower BMI, nonsmoking behavior, higher education, and better physical and mental health, consistent with previous research [6,10,11,13,14,23,40,45,53,54]. No statistically significant associations were observed with alcohol consumption in this study (only at a marginal level). Some other studies suggest a positive link between alcohol consumption and physical activity [45], such as in Henchoz’s findings [4]. A positive relationship between alcohol consumption and sport participation is sometimes interpreted as a factor of “socialization” with others. Paavola et al. [55] conducted a longitudinal study and found that smoking and alcohol use were positively correlated, while smoking was negatively correlated with leisure time physical activity. Maertl et al. [56] found a positive relationship between regular light physical activity and higher education, good coping behavior, regular alcohol consumption, and life satisfaction. However, excessive alcohol intake is a public health concern [46,47,48].

### 4.2. PA Type 1: Medium- to High-Intensity Aerobic Exercises

Men tend to prefer PA type 1 more than women, as previous studies have shown higher rates of regular PA among men [57]. Our study suggests that men tend to focus on intrinsic factors like strength, competition, and challenge, while women tend to focus on extrinsic factors such as weight management, appearance, and mental health [58]. Men’s higher intrinsic motivation leads to more consistent engagement in regular PA throughout their lives, while women tend to engage in PA more regularly as they age [59]. Women tend to prefer certain types of physical activity like dancing, rhythmic gymnastics, skating, and water sports while men prefer football, wrestling, handball, and racket sports, and these preferences may be influenced by societal norms and gender stereotypes [60].

### 4.3. PA Type 2: Low- to Medium-Intensity and Stretching Exercises

Our study found that practicing PA type 2 (such as yoga, Pilates, and tai chi) “sometimes” compared with “almost never” was associated with a higher number of visits to psychiatrists. This contradicts previous research that showed a direct association between this type of exercise and improved physical [25] and mental [61] health. However, our findings suggest that healthcare professionals may recommend these activities to enhance psychological well-being, especially for patients with depression [15,16,22,62,63,64,65]. It is possible that participants in our study who practiced PA type 2 with poorer mental health did so either because they were prescribed by doctors or decided to practice on their own.

### 4.4. PA Type 3: Outdoor Leisure Group Sport and Other PAs (Gardening, etc.)

The frequency of engaging in PA type 1 and PA type 3 had an inverse relationship with physical health (number of visits to doctors, excluding psychiatrists) and mental health indicators (visits to psychiatrists). This finding aligns with the existing literature [29,33,34,35,36,37].

There are contradictory data on the dynamics of PA with age. Generally, authors reported a general tendency for PA levels to decrease with age [66], although other authors noted the presence of an indirect linear decline in PA with age, which closer to old age may stabilize and even improve [57]. The significant relationship between higher age and regular PA type 3 (leisure outdoor activities) for both sexes, observed in the current study, could also be related to retiring and having more time for these activities.

The current study’s findings suggest that individuals with higher levels of education are more likely to engage in all three types of PA, which is congruent with the results of the Eurobarometer study [67]. This could be attributed to their greater knowledge and awareness of the benefits of physical and mental health, as well as their potentially more favorable socioeconomic status. Conversely, those with lower socioeconomic status may experience more stress and have less free time and recourse to be engaged in PA [68].

### 4.5. Study Limitations

One of the limitations of this study is the transversal design for the survey on sport activities and smoking and alcohol consumption habits that does not presume any existing causality, and we report only the existing relationship between the variables of the study. To check this, longitudinal studies would help.

Another study limitation is that we used the sum of all doctor visits as a proxy indicator of physical health. This metric may not always provide an accurate reflection of an individual’s overall health status, as some individuals may visit doctors frequently for preventative care. Furthermore, younger participants may be less likely to visit doctors compared with their older counterparts. An additional limitation of the study is the reliance on self-reporting for assessing indicators of physical and mental health. This subjective measurement approach may introduce potential bias, as individuals might misrepresent or inaccurately report these parameters.

Finally, since this was a secondary data analysis on data collected for the exploration of emotions and emotional intelligence behavior among the Belgian population [43], the initial classification of types of PAs was designed more in line with the initial purpose and we could not change it. Nevertheless, this classification helps to distinguish and display the differences in the relationship of the frequency of practicing PA type 2 with mental health (the sum of visits to psychiatrists) compared with other types of PAs.

### 4.6. Practical Implications

The results highlight the importance of promoting healthy lifestyles and PA across different segments of society, including in those with lower levels of education. Initiatives should start during adolescence, as the literature suggests that overall PA levels tend to decline during this period. Since the frequency of practicing PA was related to higher age, it is important to promote engagement in PA at earlier ages. Additionally, the findings indicate that even mild to moderate leisure activities, such as group sports or gardening, are associated with better health and a healthy lifestyle.

## 5. Conclusions

The results of this study show that women tended to practice PA type 2 more frequently, and men tended to practice PA type 1 and type 3 more frequently. A higher frequency in practicing PA types 1 and 3 was associated with better physical health (PA type 3 was also associated with mental health), lower BMI, and less tobacco consumption. In sum, the frequency of engaging in different types of PA depends on physical and mental health conditions, sex, age, and education level. Further studies are needed on how frequently prescribed the different types of PA are by health professionals for preventive (avoiding health risks) purposes or to maintain better health with age.

## Figures and Tables

**Table 1:** Descriptive statistics for the study variables.

Variables		Descriptive Statistics					
		Frequency N (%)					
	Groups *	1	2	3	4	5	6
Independent variables	Level of education	458 (5%)	1588 (17%)	2866 (30%)	2971 (31%)	1464 (15%)	213 (2%)
	BMI	179 (2%)	4103 (39%)	3538 (33%)	2841 (27%)	-	-
	General health: N of all doctor’s visits except to psychiatrists (a sum)	(0–49 visits) 2411 (25%)	(50–80 visits) 2405 (25%)	(81–123 visits) 2412 (25%)	(124–1353 visits) 2389 (25%)	-	-
	Mental health: N psychiatrist visits (a sum)	(0 visits) 8017 (83%)	(1–10 visits) 989 (10%)	(11–100 visits) 541 (6%)	(100–986 visits) 69 (<1%)	-	-
	Age group	1861 (18%)	4747 (45%)	4053 (38%)	-	-	-
	Consumption habits and PA	Frequency N (%)					
		Almost never		Sometimes		Almost always	
	Smoking (>2 cigarettes/day)	8328 (87%)		197 (2%)		1092 (11%)	
	Alcohol (>2 beers/day)	6033 (63%)		2370 (25%)		1214 (13%)	
Outcomes	PA type 1: Sport (medium- to high-intensity exercises: running, aerobics, swimming)	3986 (42%)		2867 (30%)		2764 (29%)	
	PA type 2: Sport (low to medium intensity, stretching exercises—yoga type)	8335 (87%)		854 (9%)		428 (5%)	
	PA type 3: Outdoor leisure group sport and other PA (gardening)	3536 (37%)		3494 (36%)		2587 (27%)	

Legend: * The number of groups depends on the specific variable. The groups for age, sex, and BMI are represented as follows: (a) age groups (adults, ≥18 years old): (1) <45 years; (2) 45–64 years; (3) ≥65 years; (b) BMI groups: (1) underweight ≤18.5; (2) normal weight = 18.5–24.9; (3) overweight = 25–29.9; and (4) obesity = BMI of 30 or greater.

**Table 2:** Multinomial logistic regression analysis (RRR—relative risk ratios): PA type 1 (sport of medium to high intensity: running, aerobics, swimming) with the study factors.

	Variables/Statistics	Total			Women			Men		
PA Type 1 Frequency	Factors	RRR	*p*-Value	CI (95%)	RRR	*p*-Value	CI (95%)	RRR	*p*-Value	CI (95%)
1: Almost never—baseline										
2. Sometimes	Sex	1.03	0.24	0.98–1.09	NA					
	Age group	1.03	0.40	0.96–1.12	**1.13**	0.018	1.02–1.25	0.91	0.803	0.80–1.03
	Level of education	1.07	0.003	1.02–1.12	1.12	**0.001**	**1.04–1.19**	1.03	0.339	0.97–1.10
	BMI	**0.76**	**<0.001**	**0.71–0.81**	**0.77**	**<0.001**	**0.71–0.83**	**0.74**	**<0.001**	**0.67–0.83**
	Sum of doctor’s visits (all doctors except psychiatrists)	**0.91**	**<0.001**	**0.87–0.95**	**0.90**	**0.001**	**0.84–0.95**	0.93	0.069	0.87–1.01
	Sum of psychiatrist visits	0.92	0.091	0.84–1.01	0.94	0.267	0.84–1.05	0.90	0.192	0.78–1.05
	Smoking (>2 cigarettes/day)	**0.71**	**<0.001**	**0.65–0.76**	**0.74**	**<0.001**	**0.67–0.82**	**0.67**	**<0.001**	**0.59–0.75**
	Alcohol (>2 beers/day)	1.04	0.203	0.97–1.13	1.02	0.681	0.92–1.14	1.08	0.151	0.97–1.20
3. Almost always	Sex	**1.15**	**<0.001**	**1.09–1.22**	NA					
	Age group	1.10	0.02	1.02–1.20	**1.20**	**0.001**	**1.08–1.34**	0.99	0.861	0.87–1.12
	Level of education	**1.14**	**<0.001**	**1.09–1.20**	**1.23**	**<0.001**	1.16–1.33	1.07	0.050	1.00–1.14
	BMI	**0.53**	**<0.001**	**0.49–0.57**	**0.55**	**<0.001**	**0.50–0.60**	**0.51**	**<0.001**	**0.45–0.57**
	Sum of doctor’s visits (all doctors except psychiatrists)	**0.87**	**<0.001**	**0.83–0.91**	**0.85**	**<0.001**	**0.80–0.91**	0.90	0.005	0.83–0.97
	Sum of psychiatrist visits	0.91	0.046	0.82–0.99	0.92	0.180	0.81–1.04	0.89	0.143	0.76–1.04
	Smoking (>2 cigarettes/day)	**0.59**	**<0.001**	**0.47–0.57**	**0.54**	**<0.001**	**0.47–0.61**	**0.50**	**<0.001**	**0.43–0.57**
	Alcohol (> 2 beers/day)	1.07	0.09	0.99–1.15	1.12	0.045	1.00–1.25	1.03	0.565	0.93–1.15

Legend: in bold—results with *p*-value ≤ 0.001.

**Table 3:** Multinomial logistic regression analysis (RRR—relative risk ratios): PA type 2 (stretching exercises, low to medium intensity—yoga type) with the study factors.

	Variables/Statistics	Total			Women			Men		
PA Type 2 Frequency	Factors	RRR	*p*-Value	CI (95%)	RRR	*p*-Value	CI (95%)	RRR	*p*-Value	CI (95%)
1-Almost never—a baseline										
2. Sometimes	Sex	**0.74**	**<0.001**	**0.68–0.81**	NA					
	Age group	1.02	0.730	0.91–1.14	1.02	0.756	0.89–1.17	1.04	0.741	0.84–1.29
	Level of education	1.09	0.038	1.02–1.17	**1.13**	**<0.001**	**1.03–1.23**	1.03	0.581	0.92–1.16
	BMI	**0.72**	**<0.001**	**0.65–0.79**	**0.69**	**<0.001**	**0.61–0.78**	0.80	0.021	1.02–1.06
	Sum of doctor’s visits (all doctors except psychiatrists)	1.00	0.790	0.94–1.08	0.99	0.826	0.91–1.08	1.05	0.454	0.92–1.19
	Sum of psychiatrist visits	1.16	0.021	1.02–1.31	1.14	0.087	0.98–1.32	1.25	0.071	0.98–1.60
	Smoking (>2 cigarettes/day)	0.76	0.052	0.66–0.87	0.82	0.015	0.70–0.96	**0.63**	**0.001**	**0.48–0.82**
	Consumption of alcohol (>2 beers/day)	0.99	0.983	0.89–1.12	1.03	0.706	0.90–1.18	0.95	0.586	0.92–1.14
3. Almost always	Sex	**0.72**	**<0.001**	**0.64–0.81**	**NA**					
	Age group	**1.44**	**<0.001**	**1.23–1.69**	**1.52**	**<0.001**	**1.26–1.83**	1.15	0.341	0.86–1.55
	Level of education	1.18	0.001	1.07–1.30	1.12	0.053	1.00–1.27	**1.32**	**0.001**	**1.12–1.55**
	BMI	0.54	0.041	0.47–0.63	**0.58**	**<0.001**	**0.49–0.69**	**0.44**	**<0.001**	**0.33–0.59**
	Sum of doctor’s visits (all doctors except psychiatrists)	1.01	0.914	0.91–1.11	1.01	0.864	0.90–1.13	1.02	0.809	0.85–1.23
	Sum of psychiatrist visits	1.19	0.053	1–1.42	1.19	0.091	0.97–1.46	1.16	0.404	0.82–1.65
	Smoking (>2 cigarettes/day)	**0.71**	**0.001**	**0.58–0.86**	0.74	0.010	0.59–0.93	0.62	0.015	0.42–0.91
	Consumption of alcohol (>2 beers/day)	1.14	0.070	0.99–1.32	1.19	0.053	1.00–1.42	1.07	0.589	0.84–1.37

Legend: in bold—results with *p*-value ≤ 0.001. Sex (for the total group), age group, and level of study were included as confounding factors in the multinomial regression analysis.

**Table 4:** Multinomial logistic regression analysis (RRR—relative risk ratios): PA type 3 (outdoor leisure group sport and other physical activities (gardening)) with the study factors.

	Variables/Statistics	Total			Women			Men		
PA Type 3 Frequency	Factors	RRR	*p*-Value	CI (95%)	RRR	*p*-Value	CI (95%)	RRR	*p*-Value	CI (95%)
1-Almost never—a baseline										
2. Sometimes	Sex	**1.10**	**<0.001**	**1.05–1.16**	NA					
	Age group	**1.20**	**<0.001**	**1.11–1.30**	**1.26**	**<0.001**	**1.14–1.39**	1.10	0.115	0.97–1.25
	Level of education	**1.09**	**<0.001**	**1.05–1.15**	1.10	0.002	1.04–1.18	1.09	0.010	1.02–1.17
	BMI	**0.76**	**<0.001**	**0.71–0.81**	**0.75**	**<0.001**	**0.69–0.81**	**0.77**	**<0.001**	**0.68–0.85**
	Sum of doctor’s visits (all doctors except psychiatrists)	**0.89**	**<0.001**	**0.84–0.93**	**0.87**	**<0.001**	**0.82–0.92**	0.90	0.009	0.84–0.97
	Sum of psychiatrist visits	**0.88**	**<0.001**	**0.81–0.96**	0.90	0.056	0.81–1.00	0.90	0.012	0.84–0.97
	Smoking (>2 cigarettes/day)	**0.76**	**<0.001**	**0.71–0.82**	**0.79**	**<0.001**	**0.71–0.87**	**0.72**	**<0.001**	**0.64–0.80**
	Alcohol (>2 beers/day)	1.06	0.113	0.99–1.14	1.06	0.255	0.96–1.17	1.05	0.299	0.95–1.17
3. Almost always	Sex	**1.38**	**<0.001**	**1.31–1.47**	NA					
	Age group	**1.47**	**<0.001**	**1.35–1.60**	**1.55**	**<0.001**	**1.40–1.73**	**1.34**	**<0.001**	**1.17–1.53**
	Level of education	**1.13**	**<0.001**	**1.07–1.19**	**1.13**	**0.001**	**1.05–1.22**	**1.12**	**0.001**	**0.97–1.11**
	BMI	**0.53**	**<0.001**	**0.49–0.57**	**0.54**	**<0.001**	**0.49–0.59**	**0.50**	**<0.001**	**0.44–0.56**
	Sum of doctor’s visits (all doctors except psychiatrists)	**0.80**	**<0.001**	**0.76–0.85**	**0.80**	**<0.001**	**0.75–0.86**	**0.81**	**<0.001**	**0.75–0.87**
	Sum of psychiatrist visits	**0.81**	**<0.001**	**0.73–0.90**	**0.80**	**0.001**	**0.70–0.91**	0.91	0.028	0.84–0.99
	Smoking (>2 cigarettes/day)	**0.63**	**<0.001**	**0.57–0.69**	**0.69**	**<0.001**	**0.61–0.78**	**0.78**	**<0.001**	**0.68–0.89**
	Alcohol (>2 beers/day)	1.03	0.486	0.95–1.11	1.00	0.985	0.89–1.12	1.05	0.348	0.94–1.17

Legend: in bold—results with *p*-value =< 0.001. Sex (for the total group), age group, and level of study were included as confounding factors in the multinomial regression analysis.

**Table 5:** A summary table with RRR of the most relevant factors associated with higher frequency of practicing PA (“almost always” vs. “almost never”).

Factors/Frequency of PA	PA Type 1	PA Type 2	PA Type 3
Sex	**1.15**	**0.72**	**1.38**
Age group	1.10	**1.44**	**1.47**
Level of education	**1.14**	1.18	1.13
BMI	**0.53**	0.54	0.53
Sum of doctor’s visits (all doctors except psychiatrists)	**0.87**	1.01	**0.80**
Sum of psychiatrist visits	0.91	1.19	**0.81**
Smoking	**0.59**	**0.71**	**0.63**

Legend: in bold—results with *p*-value =< 0.001.

**Figure A1:**
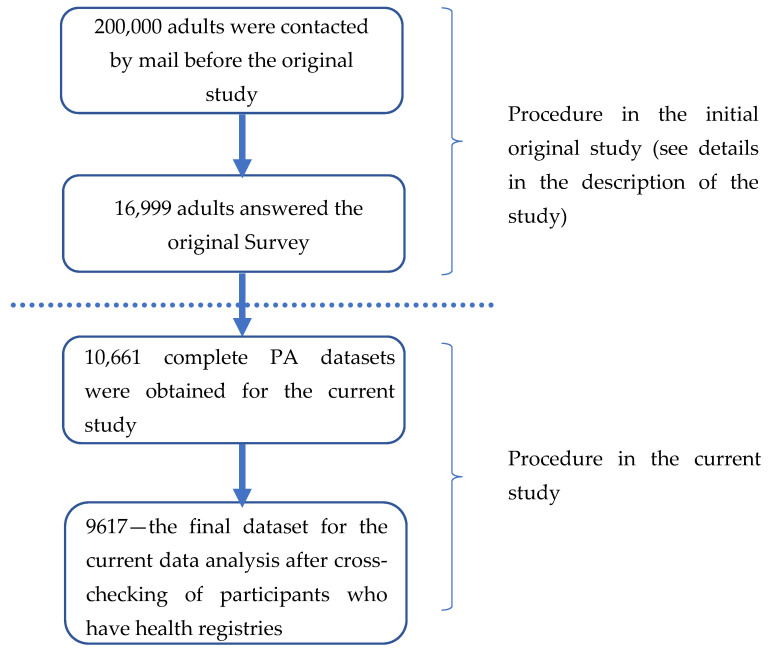
Flowchart illustrating the study sample (in the original and current studies) [43].

## Data Availability

Data are not available due not having asked permission on it in the signed informed consent.

## Appendix B. Questionnaire and Groups of Variables

### Appendix B.1. Questionnaire

#### Appendix B.1.1. Physical Activity (PA) Three Measured Types

(1) *Sport exercises: medium to high intensity*: I practice … running, swimming, aerobics, Nordic walking …) for 15 to 30 min at least 3 times per week (1—almost never, 2—sometimes, 3—almost always)
(2) *Low to medium intensity & stretching exercises* (Pilatus and yoga type): I practice yoga, stretching, Tai-Chi, Pilates or the like for 15 to 30 min at least 3 times per week (1—almost never, 2—sometimes, 3—almost always)
(3) *Outdoor leisure group sport & other physical activities*: I use some of my free time to participate in activities that maintain my condition (gardening, golf, …) (1—almost never, 2—sometimes, 3—almost always)

#### Appendix B.1.2. Healthy Life Style (Habits on Substances Consumption)

(4) *Smoking*: I smoke more than two cigarettes a day (1—almost never, 2—sometimes, 3—almost always)
(5) *Alcohol consumption*: I drink at least 2 glasses of alcoholic beverages per day (1—almost never, 2—sometimes, 3—almost always)

## Appendix C. Descriptive Statistics by Sex Groups

**Table A1:** Descriptive statistics for the study variables for women.

Variables		Descriptive Statistics					
		Frequency N (%)					
	Groups	1	2	3	4	5	6
Independent variables	Age group	1351 (25%)	2827 (51%)	1309 (24%)	-	-	-
	Level of education	193 (4%)	776 (14%)	1612 (30%)	2004 (37%)	787 (14%)	102 (2%)
	BMI	147 (3%)	2765 (50%)	1612 (29%)	963 (18%)	-	-
	General health: N of all doctor visits except to psychiatrists (a sum)	1210 (22%)	1407 (26%)	1453 (27%)	1417 (26%)	-	-
	Mental health: N psychiatrist visits (a sum)	4492 (82%)	587 (11%)	407 (7%)	-	-	-
	Consumption habits and PA	Frequency N (%)					
		Almost never		Sometimes		Almost always	
	Smoking (>2 cigarettes/day)	2312 (42%)		1664 (30%)		1511 (28%)	
	Alcohol (>2 beers/day)	4586 (84%)		601 (11%)		300 (5%)	
Outcomes	PA type 1: Sport (medium- to high-intensity exercises: running, aerobics, swimming)	2196 (40%)		2035 (37%)		1256 (23%)	
	PA type 2: Sport (low to medium intensity, relaxation and stretching exercises—yoga, tai chi, Pilates)	4824 (88%)		103 (2%)		560 (10%)	
	PA type 3: Outdoor leisure group sport and other PAs (gardening)	3894 (71%)		1143 (21%)		450 (8%)	

**Table A2:** Descriptive statistics for the study variables for men.

Variables	Descriptive Statistics					
	Frequency N (%)					
Groups	1	2	3	4	5	6
Age group	497 (13%)	1801 (47%)	1538 (40%)	-	-	-
Level of education	241 (6%)	744 (19%)	1177 (31%)	905 (24%)	653 (17%)	110 (3%)
BMI	30 (1%)	1222 (32%)	1897 (47%)	777 (20%)	-	-
General health: N GP visits (groups)	1137 (30%)	939 (24%)	889 (23%)	871 (23%)	-	-
Mental health: N psychiatrist visits (groups)	3266 (85%)	354 (9%)	216 (6%)	-	-	-
Sport and PA type, consumption habits:	Frequency N (%)					
	Almost never		Sometimes		Almost always	
PA 1: Sport (medium- to high-intensity exercises: running, aerobics, swimming)	122 (42%)		81 (28%)		91 (31%)	
PA 2: Stretching and relaxing exercises, low to medium intensity—yoga, Pilates type)	253 (86%)		26 (9%)		15 (5%)	
PA 3: Outdoor leisure group sport and other PAs (gardening)	102 (35%)		103 (35%)		89 (30%)	
Smoking (>2 cigarettes/day)	253 (86%)		8 (3%)		33 (11%)	
Consumption of alcohol (>2 beers/day)	178 (61%)		76 (26%)		40 (14%)	
